# Strong sex bias in elite control of paediatric HIV infection

**DOI:** 10.1097/QAD.0000000000002043

**Published:** 2018-10-16

**Authors:** Vinicius A. Vieira, Peter Zuidewind, Maximilian Muenchhoff, Julia Roider, Jane Millar, Margaret Clapson, Anriette Van Zyl, Delane Shingadia, Emily Adland, Rohin Athavale, Nicholas Grayson, M. Azim Ansari, Christian Brander, Claudia Fortuny Guash, Lars Naver, Thanyawee Puthanakit, Wipaporn Natalie Songtaweesin, Jintanat Ananworanich, Denise Peluso, Beatriz Thomé, Jorge Pinto, Pieter Jooste, Gareth Tudor-Williams, Mark F. Cotton, Philip Goulder

**Affiliations:** a Department of Paediatrics, University of Oxford, Oxford, UK; b Stellenbosch University, Cape Town, South Africa; c Max von Pettenkofer-Institute, Department of Virology, Ludwig-Maximilians-University Munich; d German Center for Infection Research (DZIF), partner site Munich, Germany; e Department of Paediatric Infectious Diseases, Great Ormond Street Hospital for Children, London, UK; f Department of Paediatrics, Kimberley Hospital, Kimberley, South Africa; g The Wellcome Centre for Human Genetics, University of Oxford, Oxford, UK; h IrsiCaixa - AIDS Research Institute, Badalona; i Universitat de Vic-Universitat Central de Catalunya, Vic; j Institució Catalana de Recerca i Estudis Avançats, Barcelona; k Unidad de Enfermedades Infecciosas, Servicio de Pediatría, Hospital Sant Joan de Déu, Universitat de Barcelona, Barcelona, Spain; l Department of Clinical Science, Intervention and Technology (CLINTEC), Karolinska Institute, Stockholm; m Department of Pediatrics, Karolinska University Hospital, Stockholm, Sweden; n The HIV Netherlands Australia Thailand Research Collaboration; o Department of Pediatrics, Faculty of Medicine, Chulalongkorn University; p Center of Excellence for Pediatric Infectious Diseases and Vaccines; q SEARCH, Thai Red Cross AIDS Research Centre, Bangkok, Thailand; r US Military HIV Research Program, Walter Reed Army Institute of Research, Silver Spring; s Henry M. Jackson Foundation for the Advancement of Military Medicine, Bethesda, Maryland, USA; t Department of Global Health, University of Amsterdam, Amsterdam, the Netherlands; u Institute of Infectology Emilio Ribas , Sao Paulo; v Infectious Diseases Department, School of Medicine, University of Sao Paulo, Sao Paulo; w Federal University of Minas Gerais, Belo Horizonte, Brazil; x Department of Paediatrics, Imperial College, London, UK; y HIV Pathogenesis Programme, Doris Duke Medical Research Institute, University of KwaZulu-Natal (UKZN), Durban, South Africa.

**Keywords:** elite control, HIV, infant, paediatrics, viral control

## Abstract

**Background:**

Reports of posttreatment control following antiretroviral therapy (ART) have prompted the question of how common immune control of HIV infection is in the absence of ART. In contrast to adult infection, where elite controllers have been very well characterized and constitute approximately 0.5% of infections, very few data exist to address this question in paediatric infection.

**Methods:**

We describe 11 ART-naive elite controllers from 10 cohorts of HIV-infected children being followed in South Africa, Brazil, Thailand, and Europe.

**Results:**

All but one of the elite controllers (91%) are females. The median age at which control of viraemia was achieved was 6.5 years. Five of these 11 (46%) children lost control of viraemia at a median age of 12.9 years. Children who maintained control of viraemia had significantly higher absolute CD4^+^ cell counts in the period of elite control than those who lost viraemic control. On the basis of data available from these cohorts, the prevalence of elite controllers in paediatric infection is estimated to be 5–10-fold lower than in adults.

**Conclusion:**

Although conclusions are limited by the study design, these data suggest that, whilst paediatric elite control can be achieved, compared with adult elite controllers, this occurs rarely, and takes some years after infection to achieve. Also, loss of immune control arises in a high proportion of children and often relatively rapidly. These findings are consistent with the more potent antiviral immune responses observed in adults and in females.

## Introduction

The large majority of HIV-infected children rapidly develop AIDS in the absence of antiretroviral therapy (ART) [1]. In comparison with adults, the children progress faster without ART, 50% developing AIDS within 1 year and 60% have died by 2.5 years [1,2]. Surprisingly, ART-naive HIV-infected individuals with the spontaneous capacity to maintain normal CD4^+^ T-cell counts, known as nonprogressors, are more common in children than adults and constitute 5–10% of ART-naive HIV-infected children [3–5]. The features of paediatric nonprogressors (PNP) are high viraemia in the presence of normal CD4^+^ T-cell counts, reduced CCR5 expression in the central memory CD4^+^ T-cell subset, low immune activation, and no correlation with protective major histocompatibility complex (MHC) class I molecules (HLA-B∗27, HLA-B∗57, HLA-B∗58:01 and HLA-B∗81:01) [6,7]. Many of these features are similar to those described in natural hosts of simian immunodeficiency virus (SIV) infection such as the sooty mangabey [8,9]. Adult viraemic nonprogressors (AVNP) appear to be broadly similar [10,11] to PNP but AVNP are exceptionally rare. Adult nonprogressors typically have normal CD4^+^ cell counts but low or undetectable viral loads and most express the protective human leukocyte antigen (HLA) alleles described above [12,13].

Although the nonprogressor status is more frequently seen in the paediatric population, children with spontaneous control of viraemia – often termed ‘elite controllers’ (EC) – have been scarcely described [7]. Definitions of elite controllers in HIV-infected individuals have varied but one that reached acceptance is: three or more consecutive viral loads spanning at least 1 year below 50 HIV RNA copies/ml, in the absence of ART [14–16]. Estimates of the frequency of elite controllers in adult infection range between 0.18 and 0.56% [15–19]. The immune features responsible for elite control of viraemia remain incompletely understood, although contributing factors include the spectrum of MHC class I and killer cell immunoglobulin-like receptors (KIR) molecules expressed, as well as specificity and functionality of the HIV-specific CD8^+^ T-cell response [12,13,20–22].

Here we describe a group of 11 ART-naive, vertically HIV-infected children who fulfil the criteria of elite controllers. We also describe a larger number of ART-naive HIV-infected children who achieved transient aviraemia on one or more occasions but who did not meet the elite controller criteria. Identifying these paediatric elite controllers provides an approximate estimate of the frequency of natural immune control of HIV infection in children and provides a context for anecdotal paediatric cases of posttreatment control [23–25].

## Material and methods

### Study participants

We defined paediatric elite controllers as vertically HIV-infected, ART-naive children with three or more consecutive viral load measurements over a year or more, that were below the limit of detection. The limit of detection varied according to centre and the historical period when the assays were done, and ranged between less than 150, less than 100, less than 50, and less than 20 copies/ml. ‘Blips’ higher than 1000 copies/ml were not allowed [15,16,18]. The transient aviraemia (group was defined as ART-naive, vertically infected children in whom one or more HIV RNA measurements were below the limit of detection, but without fulfilling the elite contoller criteria. The CD4^+^ T-cell count was not considered in the definition. Medical records were reviewed for clinical data, viral loads and CD4^+^ and CD8^+^ T-cell counts. All patients were diagnosed before 10 years of age and/or were born to a mother with confirmed HIV infection, supporting the notion that infection had occurred perinatally via mother-to-child transmission.

Our study involved 10 clinics caring for HIV-infected children around the world: Kimberley Hospital (Kimberley, South Africa), Ithembalabantu Clinic (Durban, South Africa), the Family Clinical Research Unit in Tygerberg Academic Hospital (Cape Town, South Africa), Instituto Emílio Ribas (Sao Paulo, Brazil), Universidade Federal de Minas Gerais (Belo Horizonte, Brazil), Great Ormond Street Hospital (London, United Kingdom), St Mary's Hospital (London UK), Karolinska University Hospital (Stockholm, Sweden), Sant Joan de Déu Children's Hospital (Barcelona, Spain), and The HIV Netherlands Australia Thailand Research Collaboration, Thai Red Cross AIDS Research Centre (Bangkok, Thailand). The cohorts selected were designed to enable us to identify any children meeting the criteria for paediatric elite controllers. We, therefore, sought large cohorts of HIV-infected children in South Africa, the country with the largest number of paediatric HIV infections worldwide, as well as in Brazil and Thailand, countries also with substantial paediatric HIV epidemics, in order to sample study populations inside and outside of Africa, respectively. Finally, we sought paediatric elite controllers among some of the smaller paediatric HIV cohorts being followed in Europe, which nonetheless are largely constituted of HIV-infected African children. Informed consent was obtained from all study participants, and for underage children, from their caregivers.

### Statistical methods

Clinical and laboratory results were described using absolute numbers, percentages, medians, and interquartile ranges (IQR). Comparisons were performed using Wilcoxon rank-sum test for continuous variables and chi-square or Fisher's exact test for categorical variables as appropriate. Age to achieving paediatric elite controller status was compared among those who maintained elite control and those who rebounded via Kaplan–Meier survival analysis using the log-rank test. We assumed a two-sided alpha error of 0.05 and used the statistical software StataSE 15.0 (StataCorp LP, College Station, Texas, USA), and GraphPad Prism Version 7 (GraphPad Software, La Jolla, California, USA). To test whether absolute or relative CD4^+^ cell counts are different between the elite controller who maintained viraemia control and those who lost, the R package lmer4 was used to produce linear mixed-effects models. Age was modelled as a fixed effect and CD4^+^ cell count (or percentage) as a random effect of each individual. *P* values were calculated using the ANOVA function in *R* to compare two models. The null model states that CD4^+^ cell count (or percentage) is proportional to age and the alternative model states that CD4^+^ cell count (or percentage) is proportional to age and viraemic control status.

## Results

**Fig. 1:**
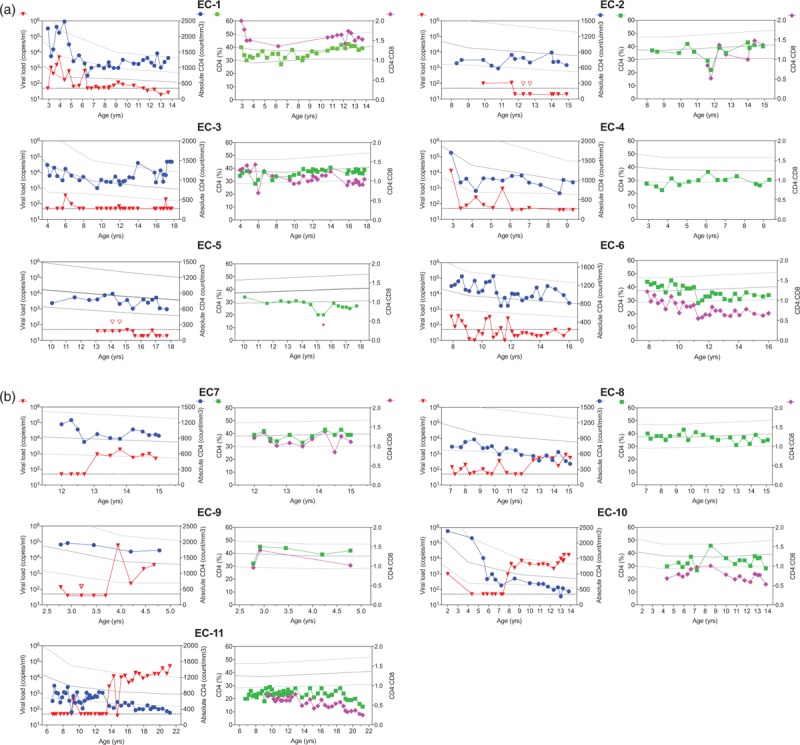
Longitudinal data of viral load (red), absolute CD4^+^ (blue), relative CD4^+^ (green), and CD4^+^:CD8^+^ (pink).

We identified 11 vertically infected paediatric elite controllers according to the criteria described above (Table 1 and Fig. 1). Ten (91%) were females. The median age at enrolment was 6.7 years (IQR 2.9–8.1years). The group included eight (73%) patients born in African countries, two (18%), in Latin America, and one (9%) in Asia.

(a) Paediatric elite controllers maintaining control of viraemia after achieving elite control. (b) Paediatric elite controllers who become viraemic after a period of elite control. Hashed-line represents the RNA HIV limit of detection of 50 copies/ml. The empty triangles represent viral load assays with the limit of detection above 50 copies/ml. The 10th, 50th and 90th percentiles are represented by the three grey lines for absolute CD4^+^ cell count and CD4^+^ percentage.

**Fig. 2:**
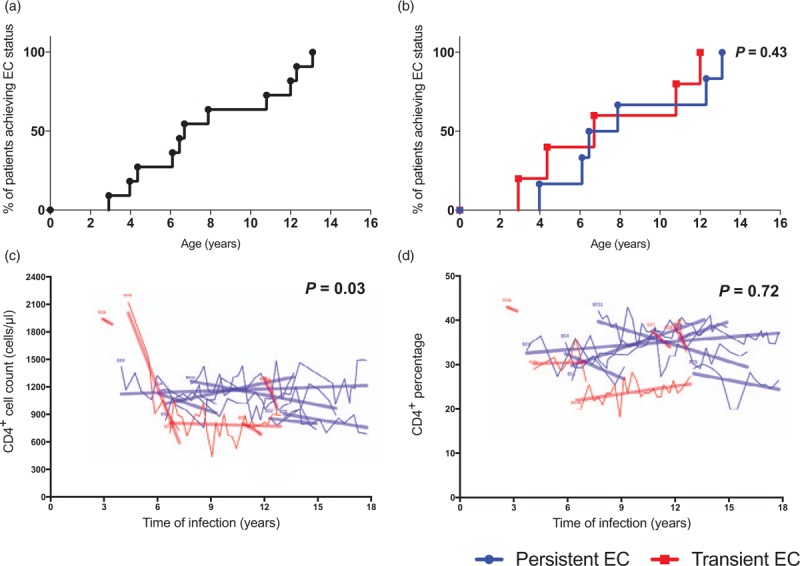
Kaplan-Meier survival analysis and mixed lineal model comparing those who maintained EC and those who rebounded.

Among the 11 study participants, seven were viraemic when enrolled but became aviraemic subsequent to enrolment, whereas four participants were already aviraemic elite controllers when enrolled (Table 1). The median age at which viraemic control was first achieved was 6.5 years (IQR 5.2–10.0) (Fig. 2a). Only three patients, EC-3, EC-4 and EC-9 achieved viraemic control before 5 years of infection. Five of the 11 children (46%) became viraemic during follow-up. The viral rebound arose at a median age of 12.6 years (IQR 7.9–13.1). The age at which elite control was achieved did not predict whether elite control would be maintained subsequently (Log-rank *P* = 0.43; Fig. 2b).

(a) Kaplan–Meier curve for age to achieve paediatric elite controller status among the 11 participants. (b) Kaplan–Meier curves for age to achieve paediatric elite ontrolle status among those who maintained elite control and those who rebounded; log-rank test was used to compare the curves. (c and d) Mixed linear model of absolute and relative CD4^+^ cell count, respectively, for those with persistent elite controller status (blue) and transient elite controller status (red). EC, elite controller.

The median absolute CD4^+^ T-cell count and percentage in the 11 patients at enrolment were 1170 cells/μl (IQR 726–1808) and 34% (IQR 31–38), respectively. All patients had an absolute and relative CD4^+^ cell count within the normal range for age during the period of elite control apart from EC-11 (Fig. 1). In a linear mixed model analysis, absolute CD4^+^ cell count was significantly higher in elite controllers who maintained control of viraemia than those who subsequently lost it (Fig. 2c, *P* = 0.03). CD4% was also higher among those maintaining viraemic control, although this did not reach statistical significance (*P* = 0.72, Fig. 2d).

**Fig. 3:**
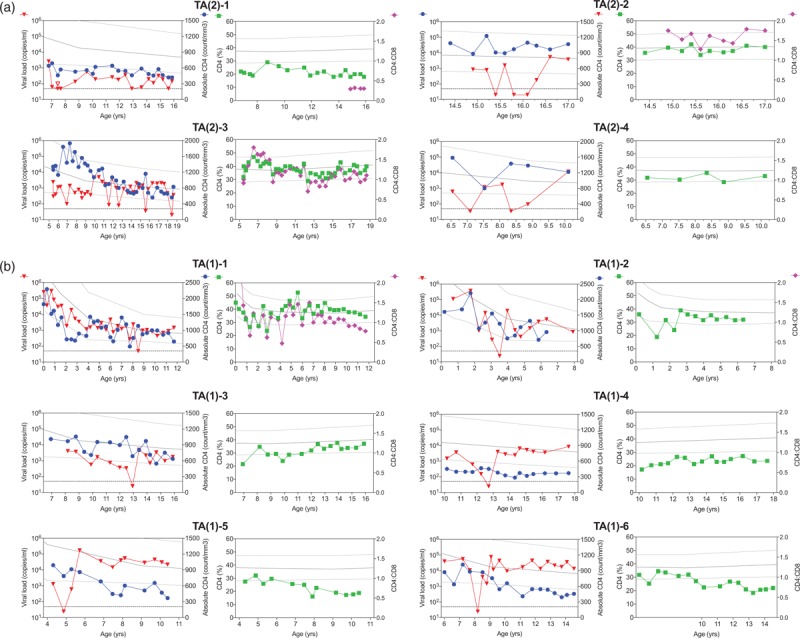
Longitudinal data of viral load (red), absolute CD4^+^ (blue), relative CD4^+^ (green), and CD4^+^:CD8^+^ (pink).

An additional group of ART-naive children with spontaneous transient aviraemia was identified within the same cohorts. Twenty-one children had at least one timepoint with viraemia below the limit of detection and four had transiently suppressed the virus for more than one timepoint, however, without fulfilling the criteria for elite controller (Supplemental Table 1, Fig. 3). Of note, transient aviraemia (2)-1 was aviraemic on three occasions, but these spanned less than 1 year. In this child, the viral load was always below 1000 copies/ml over a follow-up period of 9 years.

(a) Transient aviraemia with 2 or more viral loads below the limit of detection. (b) Transient aviraemia with one viral load below the limit of detection. Hashed-line represents the RNA HIV limit of detection of 50 copies/ml. The empty triangles represent viral load assays with the limit of detection above 50 copies/ml. The 10th, 50th and 90th percentiles are represented by the three grey lines for absolute CD4^+^ cell count and percentage CD4^+^.

## Discussion

To identify paediatric elite controllers, we applied the definition described for elite controller adults by Olson *et al.*[16] and Yang *et al.*[15], being three consecutive undetectable plasma viral load measurements spanning at least 1 year in ART-naive individuals, without viraemic spikes higher than 1000 HIV RNA c/ml. Ten cohorts of HIV-infected children were studied, from South America (Brazil), Europe (UK, Spain and Sweden), Africa (South Africa) and Asia (Thailand). Within these cohorts, we identified 11 children qualifying as elite controllers. Although four of the paediatric elite controllers were identified within European cohorts, all originated from sub-Saharan Africa. This group, thus likely represents a broad range of populations and subclades of HIV according to the global distribution: subtype B (Brazil), subtype C (Southern Africa and Ethiopia), subtype D (Uganda) and subtype CRF01_AE (Thailand) [26].

Several features of paediatric elite controllers are apparent even from this small group. The high proportion of females (91%) is consistent with the observation that adult females are five-fold more likely than males to be elite controllers [15]. Although there appears to be an increased susceptibility among females specifically to in utero MTCT [27–31], this does not appear to apply to intra partum or post partum infections [28,29], so overall, the numbers of females infected via MTCT do not exceed those of males by more than approximately 10% [28]. As in adult infection, where viral loads are typically lower in females than males [32], in some studies female children also have been shown to have lower viral loads [31,33], although this sex difference was not observed in one large study of greater than 2000 ART-naive children [34]. Among 282 paediatric slow progressors (PSP), defined as ART-naive children maintaining absolute CD4^+^ cell counts above 350 cells/μl and not meeting clinical criteria (WHO clinical disease stage 3 or 4) to initiate ART until at least 5 years of age, 59% were female [34], indicating that in female children absolute CD4^+^ cell count declines more slowly than males. Nonetheless, the numbers of female in the group of children identified here are significantly higher than that of this large PSP cohort (*P* = 0.05), suggesting that female children control viraemia better than males, as well as maintaining higher absolute CD4^+^ cell counts. This is consistent with adult studies demonstrating that the initial viral setpoint in females is lower than in males [35–37], in association with a more vigorous type I interferon response in acute infection in females [32,38].

Despite the limitations inherent in the fact that these are not birth cohorts that were studied, many of the other features of paediatric elite controllers appear to contrast with observations in adult elite controllers. First, the time to achieve control of viraemia in this group of 11 children is a median of 6.5 years, whereas in adults the time to viral setpoint is approximately 6 weeks [39]. Again, it should be noted that this estimate of the time to achieve elite control among children does not take into account the possibility that some elite controller children who achieved elite control at an earlier age were not recognized as elite controllers as a result of having lost elite control status by the time they presented; indeed, EC-1 and EC-6 experienced periods of undetectable viral load prior to fulfil the criteria of elite controllers (Fig. 1), and some transient aviraemias experienced more than one period of aviraemia (Fig. 3). Equally, other children who would achieve elite control at a future date were not included as elite controllers because they had not yet achieved elite controller status at the time of the study. Second, elite controller status appears to be more short-lived in children than in adults, in whom the median time to viral rebound among adult women is greater than 30 years [15]. The relatively common occurrence of children identified as achieving undetectable viral loads only transiently (Fig. 3, Supplemental Table 1) prompts the hypothesis that the developing immune response over childhood helps to bring viral load down over a period of years, but once sufficient selection pressure is brought to bear on the virus, in most cases it can escape. By contrast, in adults the critical battles between the immune system and the virus are waged chiefly, although not exclusively, in the first weeks of infection [40–42]. Although lack of sample availability prevents precise measurement of the viral load among the elite controller children here, certainly the number and frequency of blips of less than 1000 copies/ml among the paediatric elite controllers suggests that control of viral replication is more profound among adult elite controllers. The average viral load among adult elite controller is reportedly 1–5 RNA copies/ml [43–46]. All these above-mentioned differences between paediatric and adult elite controller are consistent with the aggressive antiviral immune response observed in adults compared with the relative tolerogenic immunity in childhood. In particular, HIV-specific CD8^+^ T-cell activity, representing the most potent arm of the antiviral immune response, does not reach adult levels until well into adolescence [47]. This explains the strong association between adult elite controller and expression of certain ‘protective’ HLA, such as HLA-B∗57 and HLA-B∗27, that mediate an effective cellular immune response against HIV [12,13,20]. Although lack of sample availability here prevents an assessment of the HLA type among the 11 paediatric elite controllers identified, HLA that are protective in adult infection have less impact in PSP, consistent with a lesser role played by virus-specific CD8^+^ T cells in paediatric infection [7].

The apparent temporal association between loss of elite controllers status and puberty in some cases (loss of viraemic control arising at 12–14 years of age in EC7, EC8 and EC-11) might seem surprising if greater potency of the HIV-specific CD8^+^ T-cell response develops through adolescence. However, in many PSP, the higher frequency and broader HIV-specific CD8^+^ T-cell responses observed with increasing age are accompanied by increased immune activation, CD4^+^ decline and, ultimately, increased viraemia [6].

The question of the prevalence of elite controllers among infected children versus adults is difficult to address when analyzing this study's data, given that the paediatric cohorts from which these 11 children have been identified have not been followed from birth. In particular, the European cohorts, that are largely constituted of children who were born in sub-Saharan Africa, are clearly selected for those who survived long enough to have emigrated. The fact that the average age at enrolment of these children is 6.7 years illustrates the point that many healthy children are likely to remain undiagnosed, especially in less well resourced countries with high adult seroprevalence where the priority is to treat adults and children with known infection and prevent disease progression. The most accurate estimates of elite controller prevalence in paediatric HIV are from Kimberley, Northern Cape and Stellenbosch, Western Cape, South Africa, in which the structure of paediatric care is such that a high proportion of HIV-infected children have been followed. In these two provinces, of 11 539 children followed, there were three paediatric elite controllers. These data would yield an estimated prevalence of 0.026% (95% CI 0–0.06%). This may represent an underestimate as healthy elite controller children are less likely to be identified in such settings. Furthermore, as it appears that maintenance of elite control status can be quite short-lived in paediatric infection, it is likely that some paediatric elite control have lost control of viraemia by the time that they present to clinicians. Conversely, other children who are future elite controllers have not achieved control of viraemia when first presenting.

The other eight cohorts studied, as described above, are relatively selected, and therefore, would represent an overestimate of prevalence. Overall, the 10 cohorts provide a paediatric elite controller prevalence estimate of 0.08% (95% CI 0.04–0.14%). Given the prevalence of elite controllers among adult cohorts of ∼0.5%, and even after considering the imperfections of prevalence estimate here of paediatric elite controllers, these data suggest that elite controller prevalence is 5–10-fold lower among infected children than adults.

The observation of transient aviraemia in a number of ART-naive children, as noted above, is not described in adult infection nor has it been noted in paediatric infection. Possible reasons why transient aviraemia have not been described in paediatric infection previously, even though within the Kimberley cohort alone, some 20 paediatric transient aviraemia were identified (Supplemental Table 1), include the fact that the HIV-infected children whose viral loads are most frequently monitored are the relatively rare ones being cared for in well resourced settings in Europe or North America. By contrast, in the more poorly resourced sub-Saharan African countries where greater than 90% HIV-infected children live, dedicated outpatient follow-up facilities, let alone frequent and regular monitoring of viral loads, are only encountered exceptionally. The mechanisms underlying transient aviraemia remain unknown. It is possible that a proportion of these would eventually become elite controllers, but in the majority, an undetectable viral load is followed by a viral load 2 log_10_ higher. One might speculate that, as the paediatric immune response becomes more effective at controlling viraemia with increasing age, so the strength of selection pressure for escape mutants intensifies. Further studies to define the mechanisms of immune control and loss are relevant to designing of strategies to achieve posttreatment control in children. However, it is of interest that among these paediatric transient aviraemias, eight of 21 (38%) are boys, a proportion similar to that among PSPs (41%) [34] and higher than among elite controllers. Numbers are too small to be definitive but these suggest the possibility that male children might be more susceptible to viral rebound once aviraemia has been achieved compared with female children.

The present study has several limitations resulting from the unstructured availability of paediatric cohorts and the absence of longitudinal data from birth. The rarity of paediatric elite controllers limits the analyses that are possible in order to describe this unique group more fully. Furthermore, paucity of samples from the majority of these individuals, many of whom are no longer being followed up, prevents a more comprehensive analysis of the potential immune mechanisms that may be operating in the children to achieve control of viraemia. However, this study does provide the first initial description of a group of longitudinally tracked paediatric elite controllers, and the striking differences that exist between these and adult elite controllers. Finally, the prevalence estimates of paediatric elite controllers is relevant in future studies of posttreatment control in children where the impact of ART or another intervention is being evaluated.

## Acknowledgements

The authors would like to thank Esper Kallás for his contribution to locate the elite controller patients in Sao Paulo, Brazil.

Authors’ contribution: V.A.V. designed research, analysed the data, and wrote the paper. P.Z., J.R., J.M., M.C., A.V.Z., D.S., E.A., R.A., C.B., C.F.G., L.N., T.P., W.N.S, J.A., D.P., B.T., J.P., P.J., G.T.W., and M.C. did the recruitment at their sites and helped with data management. N.G. and M.A.A. analysed the data. P.G. designed the study, analysed the data, and wrote the paper. All authors critically reviewed the manuscript.

### Financial support

This work was supported by the Wellcome Trust (Goulder WTIA Grant WT104748MA) and the National Institutes of Health (RO1-AI133673 Goulder).

### Conflicts of interest

There are no conflicts of interest.

## Supplementary Material

Supplemental Digital Content

## Figures and Tables

**Table 1:** Data of eleven vertically infected paediatric elite controllers.

PID	Cohort	Country of origin	Number in cohort^a^	Sex	Evidence of MTCT^b^	Year of birth	Age at enrollment (years)	Age at EC (years)	Age at loss of viral control (years)
EC-1	London, UK^c^	Ghana	400	Female	a,b	2004	3	6	–
EC-2	Durban, RSA^c^	South Africa	200	Female	a,b	2002	8	11	–
EC-3	Belo Horizonte, Brazil^c^	Brazil	644	Female	a,b	1993	4	<4	–
EC-4	Kimberley, RSA	South Africa	2501	Male	a,b	2007	3	6	10.4 (started ART)
EC-5	Stellenbosch, RSA	South Africa	9038	Female	a,b	1999	10	<10	–
EC-6	Barcelona, Spain^c^	Ethiopia	100	Female	a	2002	8	9	–
EC-7	London, UK^c^	Uganda	278	Female	a,b	2001	12	<12	13
EC-8	London, UK^c^	Zimbabwe	278	Female	a,b	2000	7	10	12.6
EC-9	Stellenbosch, RSA	South Africa	9038	Female	a,b	2010	2	2	3.9
EC-10	Sao Paolo, Brazil^c^	Brazil	500	Female	a,b	1999	3	4	8
EC-11	Bangkok, Thailand^c^	Thailand	382	Female	a,b	1996	6	<6	13.7

ART, antiretroviral therapy; EC, elite controller; MTCT, mother-to-child-transmission.

^a^Number of patients in the study cohort who did not meet the criteria for being an elite controller.

^b^a: diagnosis pre 10 years; b: mother with known HIV infection.

^c^Local clinics – the elite controller number and cohort size can not represent the HIV-infected children population in that region.

## References

[1] Goulder PJ Lewin SR Leitman EM Paediatric HIV infection: the potential for cure. *Nat Rev Immunol* 2016; 16:259–271. 26972723 10.1038/nri.2016.19 PMC5694689

[2] Newell ML Coovadia H Cortina-Borja M Rollins N Gaillard P Dabis F<ET-AL Ghent International AIDS Society (IAS) Working Group on HIV Infection in Women and Children. Mortality of infected and uninfected infants born to HIV-infected mothers in Africa: a pooled analysis. *Lancet* 2004; 364:1236–1243. 15464184 10.1016/S0140-6736(04)17140-7

[3] Mphatswe W Blanckenberg N Tudor-Williams G Prendergast A Thobakgale C Mkhwanazi N High frequency of rapid immunological progression in African infants infected in the era of perinatal HIV prophylaxis. *AIDS* 2007; 21:1253–1261. 17545701 10.1097/QAD.0b013e3281a3bec2

[4] Blanche S Newell ML Mayaux MJ Dunn DT Teglas JP Rouzioux C Morbidity and mortality in European children vertically infected by HIV-1. The French Pediatric HIV Infection Study Group and European Collaborative Study. *J Acquir Immune Defic Syndr Hum Retrovirol* 1997; 14:442–450. 9170419 10.1097/00042560-199704150-00008

[5] Paul ME Mao C Charurat M Serchuck L Foca M Hayani K Predictors of immunologic long-term nonprogression in HIV-infected children: implications for initiating therapy. *J Allergy Clin Immunol* 2005; 115:848–855. 15806009 10.1016/j.jaci.2004.11.054

[6] Muenchhoff M Adland E Karimanzira O Crowther C Pace M Csala A Nonprogressing HIV-infected children share fundamental immunological features of nonpathogenic SIV infection. *Sci Transl Med* 2016; 8:358ra125. 10.1126/scitranslmed.aag1048 PMC6087524 27683550

[7] Adland E Paioni P Thobakgale C Laker L Mori L Muenchhoff M Discordant impact of HLA on viral replicative capacity and disease progression in pediatric and adult HIV infection. *PLoS Pathog* 2015; 11:e1004954. 26076345 10.1371/journal.ppat.1004954 PMC4468173

[8] Silvestri G Sodora DL Koup RA Paiardini M O’Neil SP McClure HM Nonpathogenic SIV infection of sooty mangabeys is characterized by limited bystander immunopathology despite chronic high-level viremia. *Immunity* 2003; 18:441–452. 12648460 10.1016/s1074-7613(03)00060-8

[9] Chahroudi A Silvestri G What pediatric nonprogressors and natural SIV hosts teach us about HIV. *Sci Transl Med* 2016; 8:358fs16. 10.1126/scitranslmed.aaj1874 27683549

[10] Klatt NR Bosinger SE Peck M Richert-Spuhler LE Heigele A Gile JP Limited HIV infection of central memory and stem cell memory CD4+ T cells is associated with lack of progression in viremic individuals. *PLoS Pathog* 2014; 10:e1004345. 25167059 10.1371/journal.ppat.1004345 PMC4148445

[11] Engram JC Dunham RM Makedonas G Vanderford TH Sumpter B Klatt NR Vaccine-induced, simian immunodeficiency virus-specific CD8+ T cells reduce virus replication but do not protect from simian immunodeficiency virus disease progression. *J Immunol* 2009; 183:706–717. 19542473 10.4049/jimmunol.0803746

[12] Pereyra F Addo MM Kaufmann DE Liu Y Miura T Rathod A Genetic and immunologic heterogeneity among persons who control HIV infection in the absence of therapy. *J Infect Dis* 2008; 197:563–571. 18275276 10.1086/526786

[13] Pereyra F Jia X McLaren PJ Telenti A de Bakker PI Walker BD The major genetic determinants of HIV-1 control affect HLA class I peptide presentation. *Science* 2010; 330:1551–1557. 21051598 10.1126/science.1195271 PMC3235490

[14] Okulicz JF Marconi VC Landrum ML Wegner S Weintrob A Ganesan A Infectious Disease Clinical Research Program (IDCRP) HIV Working Group. Clinical outcomes of elite controllers, viremic controllers, and long-term nonprogressors in the US Department of Defense HIV natural history study. *J Infect Dis* 2009; 200:1714–1723. 19852669 10.1086/646609

[15] Yang OO Cumberland WG Escobar R Liao D Chew KW Demographics and natural history of HIV-1-infected spontaneous controllers of viremia. *AIDS* 2017; 31:1091–1098. 28301422 10.1097/QAD.0000000000001443 PMC5657480

[16] Olson AD Meyer L Prins M Thiebaut R Gurdasani D Guiguet M CASCADE Collaboration in EuroCoord. An evaluation of HIV elite controller definitions within a large seroconverter cohort collaboration. An evaluation of HIV elite controller definitions within a large seroconverter cohort collaboration. *PLoS One* 2014; 9:e86719. 24489776 10.1371/journal.pone.0086719 PMC3904947

[17] Grabar S Selinger-Leneman H Abgrall S Pialoux G Weiss L Costagliola D Prevalence and comparative characteristics of long-term nonprogressors and HIV controller patients in the French Hospital Database on HIV. *AIDS* 2009; 23:1163–1169. 19444075 10.1097/QAD.0b013e32832b44c8

[18] Okulicz JF Lambotte O Epidemiology and clinical characteristics of elite controllers. *Curr Opin HIV AIDS* 2011; 6:163–168. 21502920 10.1097/COH.0b013e328344f35e

[19] Lambotte O Boufassa F Madec Y Nguyen A Goujard C Meyer L SEROCO-HEMOCO Study Group. HIV controllers: a homogeneous group of HIV-1-infected patients with spontaneous control of viral replication. *Clin Infect Dis* 2005; 41:1053–1056. 16142675 10.1086/433188

[20] Fellay J Shianna KV Ge D Colombo S Ledergerber B Weale M A whole-genome association study of major determinants for host control of HIV-1. *Science* 2007; 317:944–947. 17641165 10.1126/science.1143767 PMC1991296

[21] Mothe B Llano A Ibarrondo J Zamarreño J Schiaulini M Miranda C CTL responses of high functional avidity and broad variant cross-reactivity are associated with HIV control. *PLoS One* 2012; 7:e29717. 22238642 10.1371/journal.pone.0029717 PMC3251596

[22] O’Connell KA Bailey JR Blankson JN Elucidating the elite: mechanisms of control in HIV-1 infection. *Trends Pharmacol Sci* 2009; 30:631–637. 19837464 10.1016/j.tips.2009.09.005

[23] Frange P Faye A Avettand-Fenoël V Bellaton E Descamps D Angin M ANRS EPF-CO10 Pediatric Cohort and the ANRS EP47 VISCONTI study group. HIV-1 virological remission lasting more than 12 years after interruption of early antiretroviral therapy in a perinatally infected teenager enrolled in the French ANRS EPF-CO10 paediatric cohort: a case report. *Lancet HIV* 2016; 3:e49–e54. 26762993 10.1016/S2352-3018(15)00232-5

[24] McMahon JH Chang J Tennakoon S Dantanarayana A Solomon A Cherry C Posttreatment control in an adult with perinatally acquired HIV following cessation of antiretroviral therapy. *AIDS* 2017; 31:1344–1346. 10.1097/QAD.0000000000001472 28492397

[25] Persaud D Gay H Ziemniak C Chen YH Piatak M Chun TW Absence of detectable HIV-1 viremia after treatment cessation in an infant. *N Engl J Med* 2013; 369:1828–1835. 24152233 10.1056/NEJMoa1302976 PMC3954754

[26] Hemelaar J Gouws E Ghys PD Osmanov S Characterisation W-UNfHIa. Global trends in molecular epidemiology of HIV-1 during 2000-2007. *AIDS* 2011; 25:679–689. 21297424 10.1097/QAD.0b013e328342ff93 PMC3755761

[27] Piwoz EG Humphrey JH Marinda ET Mutasa K Moulton LH Iliff PJ Effects of infant sex on mother-to-child transmission of HIV-1 according to timing of infection in Zimbabwe. *AIDS* 2006; 20:1981–1984. 16988523 10.1097/01.aids.0000247123.04703.6e

[28] Marinda E Humphrey JH Iliff PJ Mutasa K Nathoo KJ Piwoz EG ZVITAMBO Study Group. Child mortality according to maternal and infant HIV status in Zimbabwe. *Pediatr Infect Dis J* 2007; 26:519–526. 17529870 10.1097/01.inf.0000264527.69954.4c

[29] Taha TE Nour S Kumwenda NI Broadhead RL Fiscus SA Kafulafula G Gender differences in perinatal HIV acquisition among African infants. *Pediatrics* 2005; 115:e167–e172. 15687425 10.1542/peds.2004-1590

[30] Biggar RJ Taha TE Hoover DR Yellin F Kumwenda N Broadhead R Higher in utero and perinatal HIV infection risk in girls than boys. *J Acquir Immune Defic Syndr* 2006; 41:509–513. 16652061 10.1097/01.qai.0000191283.85578.46

[31] Study EC Level and pattern of HIV-1-RNA viral load over age: differences between girls and boys?. *AIDS* 2002; 16:97–104. 11741167 10.1097/00002030-200201040-00012

[32] Meier A Chang JJ Chan ES Pollard RB Sidhu HK Kulkarni S Sex differences in the Toll-like receptor-mediated response of plasmacytoid dendritic cells to HIV-1. *Nat Med* 2009; 15:955–959. 19597505 10.1038/nm.2004 PMC2821111

[33] Ruel TD Zanoni BC Ssewanyana I Cao H Havlir DV Kamya M Sex differences in HIV RNA level and CD4 cell percentage during childhood. *Clin Infect Dis* 2011; 53:592–599. 21840929 10.1093/cid/cir484 PMC3160805

[34] Mori M Adland E Paioni P Swordy A Mori L Laker L Sex differences in antiretroviral therapy initiation in pediatric HIV infection. *PLoS One* 2015; 10:e0131591. 26151555 10.1371/journal.pone.0131591 PMC4494714

[35] Anastos K Gange SJ Lau B Weiser B Detels R Giorgi JV Association of race and gender with HIV-1 RNA levels and immunologic progression. *J Acquir Immune Defic Syndr* 2000; 24:218–226. 10969345 10.1097/00126334-200007010-00004

[36] Collazos J Asensi V Cartón JA Sex differences in the clinical, immunological and virological parameters of HIV-infected patients treated with HAART. *AIDS* 2007; 21:835–843. 17415038 10.1097/QAD.0b013e3280b0774a

[37] Napravnik S Poole C Thomas JC Eron JJ Jr Gender difference in HIV RNA levels: a meta-analysis of published studies. *J Acquir Immune Defic Syndr* 2002; 31:11–19. 12352145 10.1097/00126334-200209010-00002

[38] Chang JJ Woods M Lindsay RJ Doyle EH Griesbeck M Chan ES Higher expression of several interferon-stimulated genes in HIV-1-infected females after adjusting for the level of viral replication. *J Infect Dis* 2013; 208:830–838. 23757341 10.1093/infdis/jit262 PMC3733517

[39] Robb ML Eller LA Kibuuka H Rono K Maganga L Nitayaphan S RV 217 Study Team. Prospective study of acute HIV-1 infection in adults in East Africa and Thailand. *N Engl J Med* 2016; 374:2120–2130. 27192360 10.1056/NEJMoa1508952 PMC5111628

[40] Ndhlovu ZM Kamya P Mewalal N Kløverpris HN Nkosi T Pretorius K Magnitude and kinetics of CD8+ T-cell activation during hyperacute HIV infection impact viral set point. *Immunity* 2015; 43:591–604. 26362266 10.1016/j.immuni.2015.08.012 PMC4575777

[41] Koup RA Safrit JT Cao Y Andrews CA McLeod G Borkowsky W Temporal association of cellular immune responses with the initial control of viremia in primary human immunodeficiency virus type 1 syndrome. *J Virol* 1994; 68:4650–4655. 8207839 10.1128/jvi.68.7.4650-4655.1994 PMC236393

[42] Goulder PJ Watkins DI Impact of MHC class I diversity on immune control of immunodeficiency virus replication. *Nat Rev Immunol* 2008; 8:619–630. 18617886 10.1038/nri2357 PMC2963026

[43] Hatano H Delwart EL Norris PJ Lee TH Dunn-Williams J Hunt PW Evidence for persistent low-level viremia in individuals who control human immunodeficiency virus in the absence of antiretroviral therapy. *J Virol* 2009; 83:329–335. 18945778 10.1128/JVI.01763-08 PMC2612329

[44] Dinoso JB Kim SY Siliciano RF Blankson JN A comparison of viral loads between HIV-1-infected elite suppressors and individuals who receive suppressive highly active antiretroviral therapy. *Clin Infect Dis* 2008; 47:102–104. 18494606 10.1086/588791 PMC2564994

[45] Pereyra F Palmer S Miura T Block BL Wiegand A Rothchild AC Persistent low-level viremia in HIV-1 elite controllers and relationship to immunologic parameters. *J Infect Dis* 2009; 200:984–990. 19656066 10.1086/605446 PMC3725728

[46] Maldarelli F Palmer S King MS Wiegand A Polis MA Mican J ART suppresses plasma HIV-1 RNA to a stable set point predicted by pretherapy viremia. *PLoS Pathog* 2007; 3:e46. 17411338 10.1371/journal.ppat.0030046 PMC1847689

[47] Muenchhoff M Prendergast AJ Goulder PJ Immunity to HIV in early life. *Front Immunol* 2014; 5:391. 25161656 10.3389/fimmu.2014.00391 PMC4130105

