# Supplementary material for: Strong sex bias in elite control of paediatric HIV infection
Source: AIDS. 2018 Oct 16;33(1):67–75. doi: 10.1097/QAD.0000000000002043 (PMC6750143; doi:10.1097/QAD.0000000000002043)
Supplement: Supplemental Digital Content [file aids-33-67-s001.docx]

**Supplemental Table 1**

| **PID** | **Cohort** | | | **Country of Origin** | | | **Sex** | | **Evidence of MTCT^†^** | | | |  |  |  |  |
| --- | --- | --- | --- | --- | --- | --- | --- | --- | --- | --- | --- | --- | --- | --- | --- | --- |
| TA(2)-1 | GOS, London, UK | | | Zimbabwe | | | Female | | a,b | | | |  |  |  |  |
| TA(2)-2 | Durban, RSA | | | South Africa | | | Male | | a,b | | | |  |  |  |  |
| TA(2)-3 | Stockholm, Sweden | | | Somalia | | | Female | | a,b | | | |  |  |  |  |
| TA(2)-4 | Kimberley, RSA | | | South Africa | | | Male | | a,b | | | |  |  |  |  |
| TA(1)-1 | Sao Paolo, Brazil | | | Brazil | | | Male | | a,b | | | |  |  |  |  |
| TA(1)-2 | Kimberley, RSA | | | South Africa | | | Female | | a,b | | | |  |  |  |  |
| TA(1)-3 | Kimberley, RSA | | | South Africa | | | Female | | a,b | | | |  |  |  |  |
| TA(1)-4 | Kimberley, RSA | | | South Africa | | | Female | | a,b | | | |  |  |  |  |
| TA(1)-5 | Kimberley, RSA | | | South Africa | | | Male | | a,b | | | |  |  |  |  |
| TA(1)-6 | Kimberley, RSA | | | South Africa | | | Female | | a,b | | | |  |  |  |  |
| TA(1)-7 | Kimberley, RSA | | | South Africa | | | Male | | a,b | | | |  |  |  |  |
| TA(1)-8 | Kimberley, RSA | | | South Africa | | | Male | | a,b | | | |  |  |  |  |
| TA(1)-9 | Kimberley, RSA | | | South Africa | | | Male | | a,b | | | |  |  |  |  |
| TA(1)-10 | Kimberley, RSA | | | South Africa | | | Female | | a,b | | | |  |  |  |  |
| TA(1)-11 | Kimberley, RSA | | | South Africa | | | Female | | a,b | | | |  |  |  |  |
| TA(1)-12 | Kimberley, RSA | | | South Africa | | | Female | | a,b | | | |  |  |  |  |
| TA(1)-13 | Kimberley, RSA | | | South Africa | | | Female | | a,b | | | |  |  |  |  |
| TA(1)-14 | Kimberley, RSA | | | South Africa | | | Female | | a,b | | | |  |  |  |  |
| TA(1)-15 | Kimberley, RSA | | | South Africa | | | Male | | a,b | | | |  |  |  |  |
| TA(1)-16 | Kimberley, RSA | | | South Africa | | | Female | | a,b | | | |  |  |  |  |
| TA(1)-17 | Bangkok, Thailand | | | Thailand | | | Female | | a,b | | | |  |  |  |  |
| † a: diagnosis pre 10yrs; b: mother with known HIV infection | | | | | | | | | | | |  | | | |  |
| TA: transient aviraemic | | |  | | |  | | | |  | | | |  | | |
|  | |  | | |  | | |  | | |  | | | |  |  |
